# Not *Just* Participation: The Rise of the Eco‐Precariat in the Green Economy

**DOI:** 10.1111/anti.12593

**Published:** 2020-01-24

**Authors:** Benjamin Neimark, Sango Mahanty, Wolfram Dressler, Christina Hicks

**Affiliations:** ^1^ Lancaster Environment Centre Lancaster University Lancaster UK; ^2^ Crawford School of Public Policy Australian National University Canberra ACT Australia; ^3^ School of Geography University of Melbourne Melbourne VIC Australia; ^4^ Lancaster Environment Centre Lancaster University Lancaster UK

**Keywords:** labour geographies, green economy, blue economy, precarity, bioeconomy, political ecology

## Abstract

Despite recent attention to “frontier” green economies and the governance of emerging ecosystem services, the specific division of labour in these economies has been little studied. As many such initiatives are in the global South, labour’s marginality potentially contributes to the existing precariousness of those who are more often identified as “participants”. This article examines the roles and vulnerabilities of these actors: the carbon counters, species identifiers, GIS mappers, tree planters and others operating in the shadows. We draw on current understandings of labour and precarity to examine the geographical contours of an apparent and emerging “eco‐precariat”: a socio‐economically diverse group of labourers that address the volatile demands of an ever‐expanding environmental service‐based economy. We illustrate our analysis drawing on examples from a Blue Carbon project in Kenya, ecosystem services project in the Philippines, and REDD+ scheme in Cambodia. We use these examples to theorise the nature of labour in these frontier economies and put forward a framework for analysing the eco‐precariat. We highlight the need to understand the precarity and marginalisation potentially created by this green division of labour in the provision of new ecosystem products and services. This framework contributes to ongoing analyses of labour as a central part of the green economy discourse and to larger discussions in the geographies of labour literature around the future of work in the global South and beyond.

## Beyond Participation Rhetoric towards a Geography of Green Labour

In an article in *Nature* titled “Paris climate deal hinges on better carbon accountancy”, an urgent call was made to address a critical labour strain that might befall signatory countries to the Paris climate deal (Tollefson 2016). According to its author, many countries will fall short in accounting for their carbon and greenhouse gas emissions due to the lack of skilled workers who can handle the task. Carbon accounting, said a leading climate risk consultant, is imperative to maintaining the “‘pledge and review’ programme to reduce emissions and halt global warming” and “[w]e can’t implement this agreement without building [this] capacity” (Tollefson 2016:450, 451).

This plea to create a suitable labour force to support environmental interventions and facilitate development is not necessarily new, but has intensified as of late. In fact, the “participation of locals”^1^ in community‐oriented programs has existed since the 1980s (e.g. Community‐Based Natural Resource Management—CBNRM, and Integrated Conservation and Development Programs—ICDPs). In each, local low‐paid involvement in activities such as monitoring, enforcement and project implementation was used to facilitate community buy in, establish project infrastructure and achieve conservation and development objectives. In a similar manner, the labour of local and indigenous peoples are emerging as significant to green/blue/bio‐economy (henceforth, green economy) interventions. What is new, however, is the intensified pace, scale and development of the green economy and the expanding scope of labour needed. Yet despite their contribution to the creation of financial value, the ever‐expanding local labour force often lack formal recognition, conceptualisation and appropriate compensation.

While green economy interventions have diverse foci, they share an emphasis on generating market rewards for the creation or protection of environmental services. Initiatives such as the Business and Biodiversity Offsetting Program (BBOP), Reducing Emissions from Deforestation and Forest Degradation (REDD+), Payments for Ecosystem Services (PES), and associated financial services (e.g. green/blue bonds) seek environmental protection by re‐valuing and financialising existing ecosystem products and services, whilst promising inclusive socio‐economic development through direct returns to locals. Closely related approaches include the bioeconomy, which incorporates life sciences and biotechnology to administer bioprospecting or biofuel production, and blue economy interventions that promote growth‐oriented projects in marine environments (e.g. mangrove carbon sequestration or marine minerals extraction). These represent “frontier economies” in that they redefine relationships and value within landscapes, while extending, intensifying and commodifying “new” dimensions of nature(s) to respond to global ecological crisis (Moore 2015).

The precarious nature of labour in varieties of “green capitalism” have been observed in recent scholarly work on urban waste, such as the situation of garbage collectors (Fredericks 2018; Lawhon et al. 2018) and e‐waste recyclers (Doron and Jeffery 2018; Knapp 2016). In the green economy approaches identified above, however, local engagement through the rubric of “local participation” has tended to obfuscate a clear analysis of local engagement as labour, and evaluation of the terms of these arrangements in relation to human rights, equity and sustainability. To address this gap, we ask what the green economy looks like through the lens of labour. Many such actors are usually framed by civil society and development agencies as “participants” usually by way of “projectisation” (Li 2016), and packaged under the discourse of “co‐designed” and “decentralised” decision‐making labour (Agrawal and Ribot 2000; Cooke and Kothari 2001). Yet, rarely do we hear discussions around green economy labour for those often precariously positioned actors whose labour, as carbon counters, species identifiers, GIS mappers, tree planters and other “shadow” work, is often obscured within the green economy.

We therefore see participation and labour as linked, where the former is given greater recognition than the latter in *both* green economy discourse and critiques of it. Green economy interventions often overlay existing NGO and state activities in conservation because of the growth of neo‐liberal approaches, and as conservation groups seek to mobilise new financial resources and value (Adams 2017). In this process, the transition from “local participation” to the deployment of local labour to produce value for green markets often goes unrecognised. Indeed the language of participation is often an integral requirement in finding pathways to the green economy. Up until recently, recognition of labour (definitive work) with rights and delimited time has been almost entirely absent in projects and plantation schemes, with new initiatives in the green economy only slowly giving “labour” clearer legal, political and economic recognition. In this sense, then, the distinction between voluntary and paid labour is not always clear (e.g. what types of involvement, time/wage relations, labour values and rights?) and the two are often conflated. For instance, participation can range from a (limited) voice in decision making to unpaid labour/local “contributions”. In the green economy context, participation is more often than not observed as a “performative practice that produces new socio‐spatial (and ecological) relations” (Grove and Pugh 2015:1), rather than as a site of uneven labour relations. We argue that this is a gap in our analysis, which ensures that green economy is left out of relevant discussions of labour geographies, particularly in the global South.

We aim here to explicitly examine the social and material substance of labour and various dimensions of precarity (e.g. physical, temporal, spatial, and economic) in the context of service‐based green economy interventions. This is essential if we are to grasp the geographical and social contours of labourers who contribute to creating value in the green economy (Sodikoff 2012), but may be marginalised and invisible (Neimark and Vermeylen 2017). Based on this analysis, we identify an “eco‐precariat”, which comprises a diverse, but distinctive, socio‐economic group that provides both formal and informal labour for an ever‐expanding service‐based green economy.^2^ Seeing the green economy through a lens of labour, rather than participation, uncovers the specific work that goes into “making nature legible” for the green economy and provides a deeper understanding of the different dimensions of labour precarity in the global South. Although the green economy in the global South is but one subset of the expanding neoliberal conditions giving rise to eco‐precarity, the modality of service‐based green economy interventions and the informal, precarious labour conditions they rest upon is global in scope (Dressler et al. 2010).

This article has two objectives, the first being to elaborate on the contours and characteristics of the formal and informal labour that underpin the green economy. The term “precariat” was first coined to capture the issues of inequality and unfavourable terms of employability that underpinned global service‐based economies since the 1980s (Standing 2014). We argue that these labour conditions carry over to the green economy as well. Ecosystem service markets are often sustained by an eco‐precariat whose labour, or human input, is central to securing the ecosystem “goods” or service benefits that are the focus of green economy interventions. Building on critical work in “labour geographies” (Castree 2007; Herod 1997; Strauss 2013, 2018; Yeoh and Huang 1998), we seek to examine how the “labour‐turn” can deepen our understanding of the ways in which diverse green economy interventions unfold. We take a feminist political ecology lens emphasising a renewed focus on unaccounted for, and more often than not, gendered and at times racialised labour, exposing wider issues of intersectionality, justice and precarity in green economy platforms and the uneven spatiality of labour resource access.

Second, we seek to identify the dependence of green economy interventions on a labour regime that spans varied activities to commodify “new” ecosystem products and services. While others have emphasised eco‐precarity in urban contexts (Lawhon et al. 2018), our analysis of green economy interventions centres on rural settings. We review extant critical literature and illustrative examples from published case studies on a Blue Carbon project in Kenya and REDD+ scheme in Cambodia. We also show original interview data from an ecosystem services project in the Philippines to help reinforce our claims.^3^ We illustrate our analysis using Standing’s formulation of precarious labour. Drawing on feminist political ecology, we develop a framework of eco‐precious labour that considers social relations in the green economy through a structural and labour‐oriented lens (Elmhirst 2011). This allows us to highlight not only those who lose/gain opportunities for paid work through the environmental service economy, but also whose labour becomes and can remain informal and therefore precarious. This inequity stems from the fact that green economy interventions overlay historically uneven rural political economies. This inequality and social fragmentation is explained by the precarious nature of environmental work, particularly where state and community support are weak or absent (Dressler et al. 2017). In this sense, the eco‐precariat is co‐constituted by the presence of green economy interventions. Depending on the groups concerned, these opportunities are flexible, potentially interchangeable and often unorganised but can draw effort away from resource‐based livelihoods, or incorporate educated young adults with diminishing interest in “customary” resource uses into intermittent, precarious work arrangements. In these conditions, the service‐based economy potentially reinforces poverty and vulnerability through new forms of precarious labour, while undermining pre‐existing livelihood activities outside the monetary economy. In other cases, the rural poor who exit precarious labour arrangements may simply return to land or marine based livelihoods, often maintaining the former through kin support. Either way, however, these processes ultimately contribute to uneven agrarian change.

Our focus on the intersections between labour and the commodification and financialisation of nature is highly significant for those with an interest in critical labour geographies in the contemporary era of precarious work, not just in the post‐industrial north, but globally (Munck 2013; Siegmann and Schiphorst 2016). It provides a more granular lens as to the multiple and diverse ways people engage, both formally and informally, with the green economy (Ferguson and Li 2018). We contribute to a deeper understanding of the unequal benefits and burdens of labouring in the green economy, moving beyond “participation” rhetoric and into more integrated discussions of labour rights and organisation (see Cooke and Kothari 2001; Pasgaard and Nielsen 2016). We purposefully look to engage with the field of labour geography (Castree 2007; Coe 2013), “green” organised labour (Räthzel and Uzzell 2012) and the heterogeneity of precarity (Siegmann and Schiphorst 2016; Strauss 2018). This emphasis is vitally relevant now with the adoption of eerily similar participation rhetoric by leaders of far‐right populist movements raising a set of new challenging questions around labour, and the role of the state and non‐state in a period of “post‐truth” environmentalism (Asher and Wainwright 2018; Mouffe 2016; Neimark et al. 2019).

## Eco‐Precarity in the Green Economy

Contemporary use of the term “precariat” developed in economic geography and the sociology of globalisation as a way to understand a category of labourers faced with a continually marginal existence, economic insecurity and/or social unpredictability (Standing 2012). The term gained prominence particularly as scholars were trying to make sense of “new” working‐class distinctions where flexible forms of service economies were widening and deepening capital accumulation along spatial and social lines (Munck 2013). Within this literature, the global precariat is more generally defined as a fragmented socio‐economic group who operate in a highly flexible and open labour market, and typically lack community or state support in times of need (Standing 2012, 2014). Standing notes that the “neoliberal‐turn” of the 1980s–1990s put in place a process whereby “everything should be done to maximise competition and competitiveness and to allow market principles to permeate all aspects of life” (ibid.). In effect, this meant “transferring risks and insecurity onto workers and families” (Standing 2011:1). According to Standing, a specific and invisible labour class—“the precariat”—formed during this period and came to symbolise the undercurrent of social anxiety and strife now prevalent in the new service‐oriented political economies of the global North. As Lewis et al. note, the concept of the precariat has aided an appreciation of the “chronic and deepening insecurity of social reproduction, both in the flexibilization of labour markets and the dismantling of post‐war welfare systems” (Waite et al. 2013:171, see also Lewis et al. 2015). Its empirical use has been expanded to expose deeper structural inequalities beyond just class to include gender and race (Lambert and Herod 2016; Lewis et al. 2015; Meehan and Stauss 2015), as well as socio‐political insecurities and the responses of authoritarian states to crisis (Butler 2006).

Standing’s definition of “the precariat” is not without critique, however (Choonara 2011). Most significant have been questions about whether the concept of the precariat, which seemed to emerge out of “neoliberal” market reforms and globalisation, is new or emerging as a “class” as Standing suggests. Munck (2013:751) adds that a more historical understanding of the term is needed, questioning whether northern‐focused discussions of the precariat are in reality similar to the marginality, social exclusion and “informality” documented by Latin American dependency scholars of the 1960s–1980s. As Munck (2013) points out, Marx and Engels were not comprehensive enough in recognising that various forms of precarity predated capitalism, but they did identify a “lumpenproletariat”—a “rogue” or “rugged proletariat”—less interested in revolution, but purposefully kept near capital (and markets) to fill in labour gaps in times of labour disruption. This corresponds in our study to group(s) in the green economy who somehow exist “outside” the relations of production not necessarily as formal workers, who can demand rights or benefits, but often as apolitical participants (alluding to Eric Wolf’s 2010 “a people without history”) who can be slotted in as informal casual or semi‐formal labour. In this vein, other critical observers suggest, for example, that development banks often frame the rural peasantry as “idle” surplus labour that must be absorbed informally by plantation systems for the biofuel sector (Li 2007, 2017). Munck (2013:751) also instructively highlights the French social theorist Robert Castell’s use of *travail précaire* (translated as “precarious work”) which more accurately speaks to the current “erosion of traditional work relationships and the [growing] centrality of the wage relationships” associated with a more flexible workforce. Thus, we need to be careful not to misconstrue the “rise of the (eco‐)precariat” as necessarily novel, but arising historically from the dynamics of class formation and other distinctions such as gender, race, ethnicity and sexuality, and in need of further examination. Feminist political ecology adds that our understanding of labour shifts in the green economy need to be contextualised within localised political economies and complex historical social relations, in particular uneven geographies of labour (Elmhirst 2011).

Recent scholarship has rightly identified several issues with the discourse and structural inequalities of the “triple nexus” of green/blue/bio‐economies, but the role of labour has yet to be fully grasped. Moving from the economic fringe to the mainstream, these new forms of “green capitalism” have transformed our relationship and understanding of nature into “… nothing less than a major strategy for ecological commodification, marketisation and financialisation which radically intensifies and deepens the penetration of nature by capital” (Smith 2007:17). As Moore (2015) suggests, such green “commodity frontiers” develop as capital seeks new opportunities to produce and market nature under, and within, conditions of ecological crisis. Some have observed the use of “green” services, such as ecotourism, to deflect or absorb effects of resource extraction, with the rise of green labour being proportional at times to intensifying extractive activity.^4^

Although many of these “green” products and services represent an “economy of appearances” (Tsing 2000) that might never fully materialise into any form of recognisable market, the discursive performance surrounding their construction still has tangible impacts on livelihoods (Fletcher et al. 2019; Milne and Mahanty 2019). Specifically, they shape who controls access to resources and whose labour produces financialised green environmental services, with implications for class formation and other forms of social differentiation. While the debate continues as to exactly how nature is being commodified under this ecosystem service economy (Felli 2014; Kenney‐Lazar and Kay 2017), one thing is certain: the shifting nature of work within the green economy remains relatively unexamined.^5^ Certainly, such work goes beyond the voluntary and positively spun notion of “participation” that has long been used to describe voluntary contributions to conservation and development interventions.

In response, we develop the concept of the “eco‐precariat” as a means to represent those categories of labourers who find their knowledge, skills and labour increasingly embedded into service‐based green economy platforms, particularly, but not exclusive to, the global South (cf. Apostolopoulou and Adams 2015). This includes some skilled and un‐skilled labourers and a host of voluntary personnel (foreign missionaries and non‐profit environmental tourism workers), paid local and/or casual workers, and even non‐voluntary or “hidden workers” whose gendered labour is subsumed in the production of environmental services (Standing 2009:ix).

The service‐based economy does not just create a new precarious workforce through a separation of workers and their means of production (Buck 2009). It may also solidify existing class relations of skilled and unskilled labourers, who are drawn into programmes in order to legitimise the market—this may include, GIS experts and remote sensors and government employees, many of whom must stratal on short‐term contracts between the eco‐precarity and what we, drawing on Standing (2009), call “proficians” or recognised formal environmental service work.

Illustrated by Polanyi’s “double movement”, a precarious workforce can also at times be driven towards the extremes or what we term the “hyper‐ (eco‐) precariat” (Lewis et al. 2015). Hyper‐precarity in our case may occur when locals are divorced from a means of livelihood through the establishment of protected areas and now cannot sustain productive livelihoods. This is sometimes justified by green economy advocates as acceptable, as they are creating a newly skilled labour force that can now use its skills to protect nature. The shifting nature of work may actually (re‐/de‐)skill people and draw labour away from previous livelihood activities, such as fishing, swidden farming and/or timber extraction. Indeed, some of these activities may even be stigmatised and “criminalised” under new governance regimes and economic interventions; for example, as protected areas become established, locals' status may change into “poachers” overnight (Neumann 1998). This process of stigmatisation and separation from previous livelihood activities may further serve to disconnect the precariat from familial relations, community support and natural resources. At the same time, many can also take advantage of “green moments” to secure political and economic gains and balance livelihood portfolios.

Such transformation of class relations and lives towards capitalist production in the green economy is by no means linear or without disruption (Davidson 2017). Marx (2011) in *Capital, Vol. I* describes the differences between the relationship of the capitalist mode of production and the “formal” and “real” subsumption of labour. In the case of formal subsumption, the labour process is not fundamentally modified but rather capital subsumes labour through “already existing” modes of production thereby allowing rural peasants to continue their “primitive” modes of production in parallel to taking on wage labour. For capital, however, this is limited and therefore seeks to control the relations (labour processes) and factors (technological infrastructure) setting off a secondary process called *real* subsumption where peasants are fully divorced from their means of production. Harvey (2004) calls this “accumulation by dispossession” (drawn from Marx’s primitive accumulation) to describe how people are forcefully divorced from the means of production in order to create a working class of proletariat or wage workers.

The role of such labour dynamics in the green economy is nascent but potentially significant. Market‐led global environmental governance has already had repercussions for those who have contributed to making the green economy happen. It is through discourses of participation and inclusivity where, according to Auerbuch and Negi (2009), “[t]oday, the architects of primitive accumulation feel compelled to enrol potentially dispossessed peoples in their projects”. Many times such programs target women who now carry the “double burden” of both household labour and labour expended to deliver green economy outcomes. We highlight the lack of discussion of what a fair wage is relative to the labour expended, which “market environmentalism” neglects. One might argue that the precariat is not producing value, because many green economy programs are based on service work of “non‐use". However as we have shown elsewhere, it is actually the precariat labour that creates the value, for example measuring and monitoring carbon credits (Neimark et al. 2016). Many times formal wage labour found in accounting institutions is valorised in green economy platforms without the recognition of local work.

Although the eco‐precariat appears marginal in their power over the resources that fall under environmental governance programmes and in the shaping of the green economy overall, this does not mean they are powerless or left without agency. In fact, local resource users have a history of navigating work opportunities within the green economy. However, such labour agency must be historicised (Herod 1997), not just through class analysis, but also through the lens of social, cultural, gendered and racialised structural violence to understand the importance of differentiating labour from participation (Elmhirst 2017; Werner 2015). It is because of this complexity that we feel it is essential to re‐focus on how labour is analysed, and move beyond notions of participation in green economy platforms in the global South.

## Labouring in the Shadows: From Proficians and the Hyper‐Precariat to the Criminalised and Dispossessed

For the past 25 years, post‐industrial economies have shifted towards a more financialised and service‐based workforce, and a transformation of work by automation and specialisation. This has forced many to rethink different categories of labourers and how wage relations can be restructured through new transnational and virtual networks (World Bank 2019). The beneficiaries and those bearing the burdens of this new division of labour in the global North have been discussed at length (Castree et al. 2004; Urry 2014), and more recently taken up by those concerned specifically with precarity (Strauss 2013, 2018). A useful thread of literature makes an important analytical and empirical distinction between “work as the labour processes” and “labour power or the capacity to work” (Thompson 1989). Gill and Pratt (2008) add that such distinctions are affected by the materiality of changing workplaces (e.g. creative industries) and that these changing economies fit into workers' livelihoods and their “autonomous” agency to adjust and/or resist (Carswell and De Neve 2013; cf. Herod 1997). New divisions of labour and problems of fragmentation and heterogeneity of precarious workers may correlate with the difficulties of organising resistance (Räthzel and Uzzell 2012; Siegmann and Schiphorst 2016; Strauss 2018). Yet, some of the blame has been attributed to new extractive and remedial technologies that depend more on algorithms and less on human labour. Nevertheless, these discussions highlight even more that much of the concern of “the future of precarious work” have been in post‐industrial sites in the global North, and very little concerning the labour organising and resistance of labour in green economy interventions.

We know, however, that these shifts are not isolated to the global North. Neither is the rise of the eco‐precariat just a case of “global South informality”, where the finances and policy emanate from sources far removed from the site of intervention. In fact, recent changes in the green economy have forced a rethink of people’s participation, where people, landscapes and oceans are managed in discrete but econometric and techno‐scientific units over larger areas (e.g. estimated blocks of carbon using LIDAR remote sensing). The implications of automation are far reaching, especially since service industries have been shown to have intense disruptions for those already in precarious working conditions (cf. Meehan and Stauss 2015). The question arises of how such automation further marginalises green economy workers, especially women’s labour, since these technologies depend upon skilled professionals who can fill the role of bureaucrats, mid‐level managers and technicians to conduct these accounting and reporting tasks—many of these filled by male professionals (see also MacDonald 2010; Sklair 2001).

Less understood are those operating in the shadows to steward the ecosystems services that are marketed by this service sector. These workers seem to fall through the cracks, particularly where their labour is hidden under a guise of “inclusivity” or “participation” discourse, which consists of work opportunities that are casual, temporary and for the most part highly precarious (see Table 1). For instance, the FAO’s new Blue Growth Initiative (RI‐BG) states that “ … member countries across a range of outcomes [sic] will … develop inclusive and gender sensitive equitable aquaculture and fisheries value chain” (FAO 2014). And quoting from the Seychelles government, which has adopted the RI‐BG platform, “[t]he Blue Economy is fundamentally about social inclusion”. In a gender‐focused analysis, the UN report discussed how “… women’s participation in inclusive, sustainable and green growth can propel the growth of a green economy. Women are consumers, they are also workers and producers, and in this context they play a crucial role in benefiting the growth of a green economy and in reaping the benefits from it …”.^6^ The UN states that “an inclusive green economy is one that improves human well‐being and builds social equity while reducing environmental risks and scarcities”.^7^ Yet, with all the talk about “socially inclusive” and “gender sensitive” development, it is quite ambiguous exactly how labour fits, particularly labour rights—and forces us to think more deeply about how different labour needs are actually met as these projects develop on the ground.

**Table 1:** Main Categories of Actors and their Labour for Operation of the Green Economy

Category of labour	Scale of operation	Work tasks and structural position	Type of work	Degree of local natural resource reliance and green economy market integration	Forms of payment or returns for labour
*Proficians*					
Managerial class	Transnational	Highly skilled workers in international agencies; nationally based expatriates; consultants; other highly skilled roles	Accounting and financialising ecosystem services; surveying; training; data management; technical services; remote sensing	Low resource reliance and high market integration	Salaried; contract‐based
State/tertiary sector technicians	Transnational/national	Highly skilled nationals; nationally based expatriates; international researchers	Accounting; data management; technical services; research; training; policy design and implementation	Low resource reliance and high market integration	Salaried; rolling consultancy contracts; grant‐based employment
National and voluntary NGO managers	Transnational/national	Medium‐skilled nationals; expatriates working with national NGOs	Community consultation; project planning and implementation; technical services. Forest rangers, and the bureaucrats created to monitor, measure and account	Low resource reliance and high market integration	Salaried; contract‐based; voluntary
*Precariat*					
Professionalised casual workers	Local	Medium/low skilled workers securing the production of ecosystem service (e.g. controlling access)	Local leaders; patrollers; ecotourism operators	Medium resource reliance and medium market integration	Casual work (short‐term contracts or day labour); immediate or deferred payments; possible other incentives/benefits
Hyper‐precariat	Local but also sometimes found to be dispossessed or criminalised	Restricting activities, for example, through licensing, to produce ecosystem service (e.g. restricting livelihood activities)	Ex‐fishers/pole cutters; temporary local NGO workers; community groups, students, volunteers	High resource reliance and low/medium market integration	No contract; “invisible”; no prospect of financial benefits
Dispossessed	Usually migrant or local labour due to either being deposed or livelihood criminalised	Small‐scale fishers; swidden farmers/horticulturalists/hunters/non‐timber forest product harvesters; guides, porters	Local eco‐precariat negotiates agrarian labour and ecosystem services labour through social relations, kin	Very high resource reliance and low market integration	Made invisible through the social stigma attached to old livelihoods, criminalising certain activities or portions of the existing workforce

Our table describes several areas of labour that are necessary to the functioning of the green economy. In the top part are the transnational managerial class, technicians and project consultants and managers who make up the visible spectrum of labour. This group is high skilled and most apt to benefit from an expanding green economy—what Standing (2009:ix) calls “proficians”. In the lower part of the table are three categories that form an emerging body of “eco‐precarious” labourers that are respectively professionalised casual workers, hyper‐precariat, and dispossessed. These heterogeneous groups encompass a diversity of workers who are incorporated into the green economy platforms in different ways—some voluntarily and others out of desperation. The majority of this work remains hidden under the veneer of participation and inclusion in green economy discourse.

We see this secondary (eco‐)precarious class in three ways: (1) a “produced” class of workers *professionalised* to conduct labour tasks that are low‐paid, temporary and flexible; (2) a *hyper‐precariat* of unpaid local and other workers (e.g. international volunteers); and (3) workers who are *dispossessed* or highly mobile and able to fill labour opportunities. In addition, attached to these different classes of labour are varying degrees of resource reliance and market integration, which must also be explicit as they underlie how and to what degree these labourers are subsumed under green economy platforms. Hence, more broadly, paid local workers on contracts are typically better integrated into the market/service sector and less likely to be full‐time farmers or fishers; the “hyper‐precariat” at times may have diversified labour roles that “bridge” service and resource uses; and last, those who are dispossessed are most resource reliant and easily criminalised as they remain on the socio‐economic margins.

### Professionalised Locals on Flexible Short‐term or “Casual” Contracts

Beyond the participation and inclusion discourse put forth by proponents of the green economy, the eco‐precariat is often targeted through payment to “leave nature alone”, so transforming their livelihoods, but may then have their labour and local knowledge drawn on to conserve ecosystem services. This potential or actual change in their relationship to nature is important because these same groups are highly reliant on the resources that they are now being “charged” (and even sometimes paid by green economy organisations) to protect. Thus, the resource‐reliant eco‐precariat identifies the environmental service through their labour (and knowledge), but is then a disciplined recipient of it as well. While sometimes the labour is recognised, depending on the skill set of the worker, much of the less skilled is rendered invisible through marginalisation of these same workers. Indeed, the effects which the labour restructuring has on their livelihoods, identities and social relations go unrecognised and uncompensated. As nature is now protected under some form of newly financialised service (e.g. green bond), their livelihoods dramatically change in the process, as do their daily interactions.

As an example, the blue carbon project in Kenya is run by the government‐registered community‐based organisation “Mikoko Pamoja Community Organisation” (MPCO) that, through the sale of carbon credits, funds mangrove conservation and other community projects in the local area. The Mikoko Pamoja project has been generally recognised as successful at restoring and conserving mangroves through community participation, with critical academic analyses focused on how improvements in governance can address an “implementation gap” (Kairu et al. 2018:74; see also Cleaver 2012; Lovell and MacKenzie 2011). However, this project can also be used to illustrate how new green/blue economy platforms are harnessing casual labour within the discourse of “local participation”. The receipt of funding to MPCO through carbon credits is contingent on the project demonstrating “good governance” and increases in levels of “carbon sequestation”, requiring a number of changes to previous forms of labour and work. Prior to the project launch in 2010, a number of local residents were engaged as subsistence‐based fishers and mangrove pole cutters (Rönnbäck et al. 2007); others, as a forestry requirement for community participation (Government of Kenya 1994), were employed by a government research organisation as manual labourers in mangrove reforestation. The new Mikoko Pamoja project altered labour relations in two ways. First, in an effort to protect the forest, harvesting was excluded from the project area to all but a limited number of official harvester licencees, and communities enlisted to self‐enforce the protection of mangroves from “illegal” harvesters. This formal subsumption of labour reduced the number of legal subsistence fishers and pole cutters in the area. Second, because the project needed to establish a system of monitoring and reporting mangrove restoration and growth, they employed a new set of labourers to map, delineate and monitor mangrove growth; and, using remote sensing and GIS technologies, to measure progress and account for the carbon captured through their mangrove restoration and protection activities.

This is reminiscent of Lansing’s (2012) example of “what type of work” takes place in order to identify spaces and nature used to create value in the green economy. He describes the performance of different “collective agents” and their “calculative practices” which contribute to the combined agency of making carbon exchangeable (Lansing 2012:207). An assemblage of scientists and local indigenous leaders “participate” through the adoption of GPS devices to map carbon‐sequestering trees in Costa Rica. In another example, Lovell (Lovell and Ghaleigh 2013; Lovell and MacKenzie 2011) describes specific groups who count carbon and who are tasked with activities which may lead to the construction of a carbon market. Often the measuring and monitoring of carbon projects is not being done by local labour at all, but increasingly “estimated from afar” through advanced airborne or satellite remote‐sensing data, Lidar and algorithm‐based “allometric models” (Saatchi et al. 2011). Other good examples of automated tools disrupting green economy work include, in forest conservation, the use of acoustic monitoring, camera traps, barcoding and drone technologies; and, in marine monitoring, robotics. These are used to survey forests and marine species, and who is entering forests and fisheries, respectively, with the adoption of “big data” to enhance green economy work. While the debate goes on as to which is the best type of technology to do this work, there is little discussion of how these “disruptive technologies” may contribute to local precarity. For example, drones or species estimates using robotics, may reduce labour opportunities for local ecologists, botanists and other conservation workers, who were trained to identify and create inventories of species. Furthermore, there is also the reality that these new technologies are meant to monitor those same workers who are now either “jobless” or displaced from entering protected areas, both marine and terrestrial (see Sandbrook et al. 2018).

Neimark and Wilson (2015) are quite specific about how botanists’ labour is used to collect and categorise biodiversity for large NGOs’ drug discovery programmes, and how this knowledge is later re‐appropriated for biodiversity‐offsetting schemes. This work discusses work roles involved in scientific labour for bioprospecting, and how technologies, such as geospatial technology and high‐throughput screening—some that are essential for drug discovery—remain out of reach for many Malagasy scientists. Scientific labourers in Madagascar lean on what amounts to “coerced collaboration” because they lack the advanced technology to conduct basic lab experiments and thereby “sell their labour” to international bioeconomy platforms. Their “participation”, however, is key in the selling success of green economy projects even though it reinforces a scientific class‐system that consigns such personnel to the position of technical staff in their own labs.

This builds on Sodikoff’s (2012) examination of the conservation workers in Madagascar demonstrating how low‐paid local conservation workers must maintain a balance between their day jobs of protecting local environments for international NGOs, which includes keeping friends and neighbours out of forests, while maintaining subsistence livelihoods through shifting cultivation—the bane of conservationists due to its destruction of primary forests. Conservation workers in Madagascar are in such marginal working conditions that it undercuts conservation efforts, as many conservationists by day need to feed their families through swidden cultivation at night (see Sodikoff 2012). Yet, such examples from the Mikoko Pamoja project in Kenya show that significant slippage takes place between those whose paid labour is recognised within “participatory forest management” projects and the critical need to sharpen our focus on uneven labour relations within such projects (Kairu et al. 2018).

### Hyper‐Precariat: Unpaid Locals

The Mikoko Pamoja project mentioned above is a partnership with KMFRI (the Kenyan governmental fisheries research organisation) and involves training for forest managers and rangers. Yet the project also collaborates with Earthwatch (https://earthwatch.org), a group that combines “volunteer opportunities for individuals with scientific research expeditions to conserve wildlife and the environment”. This “citizen science” project in the form of “volunteer tourism” fits with much of the casual labour arrangements where volunteers do the bulk of the monitoring and locals become participants as either beneficiaries or in unskilled labour roles. Another example of this is the work of “scouts” or local casual workers who receive training and help monitoring, but are not contracted—instead they do this work in expectation of compensation at a later stage (i.e. in the form of becoming employed as rangers or becoming licensed harvesters).

Many smallholders or fishers, for example, whose labour is drawn in support of measuring and protecting ecosystem services, already live in marginalised environments with limited economic opportunities and declining ecological conditions which constrain livelihoods. By being pulled into such green economy labour arrangements their time is often taken away from varied livelihood activities and domestic responsibilities, requiring that they renegotiate household divisions of labour (relying, for example, on grandparents to mind children and fields while they measure trees or weigh seaweed to sequester carbon). This transition does not apply to everyone in the localities targeted by green economy interventions, however, as many may still rely on and continue to use natural resources (albeit with new risks). In these settings, interventions may be unsuccessful in changing resource use in the intended ways, such that the protection of ecosystem services functions as a façade (Milne et al. 2019; Neimark et al. 2016).

Market‐driven interventions are set up in order to integrate those into the global market, albeit with mixed results. Lewis et al. (2015:584) describe a form of “hyper‐precariat” for those who express visceral feelings of “un‐freedoms” that arise with their predicament. In the southern Philippines, the indigenous Pala’wan have recently been incorporated into the casual labour pool of big international NGOs. In particular, the Philippine chapter of one NGO has been drawing on the labour of Pala’wan swidden farmers to help sustain forests as natural capital in the Mount Mantalingahan Protected Landscape (MMPL) of Palawan Island. In 2008, the NGO and partners estimated the value of the broader environmental services of MMPL, including timber, soil, watershed dynamics and marine biodiversity, at over US$5 billion in total (Protected Area Management Board 2010:17). Apparently, this value far exceeded the economic value of mining (at roughly US$3 billion) and other resource uses such as upland agriculture. By using a “total economic value” framework, the NGO and partners asserted that “at a 2% discount rate, the benefit from water for domestic agriculture and fishery was highest at P68.092 billion followed by the benefit from carbon sequestration, valued at P33.788 billion” (Conservation International et al. 2008:2).

In the MMPL, Pala’wan Palawan chapter of the NGO soon needed cheap, indigenous labour. Offering indigenous Pala’wan various incentives to become part‐time forest stewards in the MMPL, the NGO established a Community Conservation Agreement (CCA) with the local Pala’wan Tribal Council in the remote village of Maracunan. Similar to Sodikoff’s (2012) case in Madagascar, the families comprising the tribal council and other villagers who had entered the CCA depended heavily on swidden. The NGO disapproved because of the perceived environmental costs and because the organisation was conditioning farmers to police and abandon swidden. Emerging as a hyper eco‐precariat, these Pala’wan families were labouring to protect old growth landscapes by simultaneously criminalising and undermining their main source of subsistence, swidden agriculture, on customary lands (Dressler et al. 2018).

In practice, the CCA Model promoted inducing local conservation strategies to address the opportunity costs of foregoing destructive resource uses, including the lost income otherwise generated from the conversion of forest to swidden agriculture. A key aspect of the CCA agreement between the NGO and Maracunan was that farmers would become deputised as “community rangers [who] in coordination with the local peoples would monitor the area to ensure old growth forest and threatened species would not be cleared for swidden and harvested for consumption or sale, respectively”. In return, the NGO offered PHP 1000 (US$20) per month for rotating patrols (usually shared between two people) and four pesos per sapling planted in old swidden fallows.

Those Pala’wan who laboured for the NGO suggest the funds were insufficient and that cash payments for the planting efforts were not forthcoming. Instead, the Pala’wan were paid in bags of rice. Distressed by their inability to return to clear and burn fallows planted with NGO trees, the labourers resisted in their way by simply planting all remaining saplings into one derelict swidden fallow. When asked about the NGO’s project and its tree planting initiative, a farmer noted with ambivalence:

I can’t disagree and I cannot agree … we just follow along … I mean we received chickens, one lived the rest died because of a stomach infestation. So they say “if you don’t cut down the old forest, we will give you a chicken”. But we were also told to plant a lot of trees in our *uma* [newly cleared] fields. I was given 400 saplings and was supposed to be paid 4 pesos per tree. So we planted many of them in our fields but after a while we had to stop, and then planted all the trees in one area only! [outside of the *uma*] Our *panglima* suggested we do this … He said that if we plant all these trees in every *kaingin* [swidden], in the future we won’t have any area for kaingin. So we really don’t have a choice but to open in *giba* [old growth].

In many respects, then, the NGO’s campaign to use Pala’wan farmers as cheap labour to defend natural capital led to predictable outcomes. Rather than labour to reforest without pay, these farmers simply continued to clear and burn secondary forests (in the same areas the NGO wanted to conserve them).

### Dispossessed, Criminalised, and the Highly Mobile Filling Gaps in Labour Opportunities

The last group comprises those whose livelihoods are criminalised and/or have been dispossessed, evicted or lost access to their land or resources and are now seeking alternative forms of livelihood. In essence, these are the rural poor who create value for nature by curbing or abstaining from fishing or farming, either voluntarily through coercion, or because their livelihoods have been criminalised through new management policy. This goes beyond just divorcing many from their livelihoods or resources, but also challenges their identity (e.g. as fisher folk) and reconfigures social relations as the division of labour shifts under new resource access. It may, in some cases, also influence local moral economies of gaining and maintaining needed social support. As Sodikoff (2012) notes, those who choose to work for green economy programmes need to strike a very delicate balance with their friends and neighbours on whom they are now charged with enforcing environmental rules. For example, unregulated, small‐scale fishers are counted in national statistics alongside illegal fishers, and have become a recent target in efforts to “secure” the blue economy sites from overexploitation. In contrast, upland swidden farmers are often not included in national census statistics but continue to be monitored and managed by non‐state actors who enrol them as forest guardians or labourers while criminalising their agriculture and subsistence base (Fletcher et al. 2019; Mertz et al. 2009). As observed above, criminalisation does not imply full compliance with these rules, but can constrain local opportunities in significant ways. Without labour rights, the hyper‐precariat has no local recourse.

These are the many unaccounted for, or unpaid, labour tasks that are vital to the success of the job of “making nature visible in a way that capital can see it” (Robertson 2012). These roles are often performed under the promise of paid work in the future, or a belief in future reward. Indeed, these engagements can at times lead to paid and even permanent work as individuals negotiate their position and power. While not mutually exclusive, all of these in one form or another constitute another form of eco‐precarity. For instance, in both the voluntary carbon market and emerging arrangements for REDD+ under the UN Framework Convention on Climate Change, evidence that local “labour” providers (although not framed as such) are consenting participants has proved essential. A skilled cadre of technically trained workers has emerged to undertake the production of the necessary evidence of Free Prior and Informed Consent (FPIC) (for the voluntary market) and to comply with “Safeguard Information Systems” that are developing for REDD+ under UNFCCC. FPIC represents a critical moment in the creation of a valuable, trusted and fungible commodity (Milne and Mahanty 2019).

As green markets (e.g. for forest carbon) are uncertain and volatile, it matters when and how labourers are compensated for their efforts. In a case study project in Cambodia (Mahanty et al. 2015), the labour of project development and consent‐gaining received immediate recompense from donors, civil society and/or government. Yet those responsible for securing the “production” of carbon on the ground, particularly through altered land use practices, had to wait for the credits to be sold, with high levels of uncertainty about how much would filter through to the village level after government and other actors had taken their share. Thus, “knowledge brokers” or proficians were well positioned to gain significant and immediate revenues from forest carbon markets, while local ecosystem service “producers” had to wait. This temporal inequity translated to a distributional inequity as well, given that payments from carbon buyers were uncertain and a significant share was channelled to national state agencies.

This does not mean, however, that local actors are without agency or understanding in relation to these skewed arrangements. As one villager stated:

I participated twice in the REDD meetings and know that REDD is about carbon and selling carbon to the factory countries. The workshop trained people to do conservation and prevented them from clearing the land—we were strictly banned from clearing land and cutting wood. But we asked the question why don’t they stop the powerful men who have guns, the high‐ranking people in this province and the rich who use money to hire people to destroy the forest—they are the ones causing deforestation, not the people. Until now we haven’t yet got the answer from the NGO. (Villager Interview, 12 December 2013)

With his statement, the villager revealed his suspicion of future benefits in return for local restrictions in resource use, as well as consciousness about the inability of REDD+ to address the political economy of forest loss. The outcome on the ground has therefore been weak local support for the deferred and unpaid benefits of ecosystem service provision, which ultimately poses risks to the scheme (Milne et al. 2019).

Similar issues exist for those fisher folk under new “blue economy” monitoring and “transparency schemes”. A new scheme, Global Fishing Watch, has recently been launched in collaboration with Google Earth to track registered fishing vessels, contributing to ongoing efforts to control IUU (illegal, unregulated and unreported fishing). While efforts in the blue economy to clamp down on IUU fisheries (EJF, FAO) and exploitative labour conditions has been around for some time, the problem of lumping unregulated and unreported fisheries together with illegal fisheries now especially has the effect of disproportionately criminalising small‐scale and informal fisheries in blue economy schemes. Small‐scale fisheries comprise up to 50% of global catches, most of which is unreported (Froese and Pauly 2015), and 90% of which involves fishers from low income countries (Mills et al. 2011). Strategies to combat IUU which focus on monitoring commercial fisheries, many of which are registered and licensed, risks consolidating fishing into fewer hands, and further marginalising small‐scale fishers through new monitoring networks. Meanwhile, it is transnational skilled surveillance workers and technology specialists, together with global companies (e.g. Google), who supply the monitoring tools responsible for monitoring fishing to “reap the benefits” of the blue economy.

## Articulating the Eco‐Precariat as a Future Research Agenda

A critical reading of green economy discourse tells us that “technical staff work, but local people participate”. Since the green economy has commodification as a central tenet, however, we consider it more appropriate to reframe this analysis to one based on labour. Examining shifts towards service‐based environmentalism through a lens of eco‐precariat, rather than “participation”, allows comparison with parallel studies of labour precarity in more urban settings in the global South. This article provides one way of thinking about how to move beyond participation towards a discussion of uneven labour relations both in, and beyond, the green economy. Building on Standing’s use of the precariat, we place a particular attention on those who are most marginal, or the eco‐precariat—which provides both formal and informal labour for market‐environmentalism in many forms, from the casual contracts for field guides in bioprospecting to tree planters for REDD+ decarbonisation programmes. We argue that critical scholarship needs to pay particular attention to how labour is subsumed in the green economy and that our analysis not only adds to important debates on the “future of work” (World Bank 2019), but also alternatives, such as a living wage (Adams and Neumark 2004) and de‐growth (Hickel and Kallis 2019)—although the latter, according to Barca (2019:209) and we agree, has yet to provide a clear way forward to address the “fundamental political problem” of fragmentation and alienation of workers.

### What does the Green “Gig” Economy Look Like in the Global South?

The green economy is a topic of focus because it is representative of larger processes of “economic” activity more broadly. Its very nature of flexible formal/informal labour needs, short‐term subcontracts between institutions and agencies, and overall movement towards service‐based casual work, is both vital to its legitimacy and efficiency (i.e. making it legible for capital, but also ticking the rhetorical “box” of inclusion) and provides a key space to investigate what we see as a labour turn in studies around the green economy. While our analysis does focuses on rural settings, certainly more scholarship is needed around eco‐precariat and precarious green economy labour in urban spaces and the further implications in terms of intersectional relations, most namely gender and race. All critical issues that we think are now open to further analysis using the eco‐precariat framework.

Similar to other types of informality, and work precarity, such as the “gig” economy, the green economy is based on an ever‐increasing “shell of services” which do not need long‐term employees, and thereby circumvent all of the often legally defined labour services (e.g. health care, social security) that come with employment in other more established economic sectors (Castree et al. 2004; Ross 2008). Rather, for the most part in rural green economy setting, it first needs locals to “participate” mainly by “stepping aside” so conservation or development agencies can take over the more technical resource management and governance roles while also providing traditional extraction some “green cover”. In establishing itself, however, a programme and projects also need casual precarious labour to fill in for tasks like planting and patrolling (Dressler et al. 2018; West 2006). It is in our minds a similar modern‐day service economy, without the need for long‐term labour. The resource‐reliant eco‐precariat are never fully integrated into any formal labour regimes—because they do not exist in the green economy’s economic models or they are built on informality and hidden work. In other cases, the eco‐precariat may exist in the ambiguous middle ground of donor aid, conditions and national laws—liminal state‐non‐state spaces—wherein it is easier to criminalise than uphold their rights. Precarious citizenship produces precarious labour in the green economy. Sometimes, however, the service is transnational in shape as its commodity chain extend globally, for example e‐waste or carbon. Rather than a global South eco‐precariat, maybe we need to begin thinking about a “global eco‐precariat”, one that follows precarious labour back and forth across the global North and South and is careful to include the contextual political economies of place‐based geographies. Furthermore, there is a lot of potential for further focus on eco‐precarity and de‐growth—seek out new opportunities and engage the limits around questions of living wage, and other lingering issues around what “de‐growth” may look like in the global South in regards to labour precarity.

### Why is the Green Economy So Fragile?

We argue that some of the weakness and fragility of the green economy rests in part on its reliance on an informal, often hidden, workforce without legislated rights, structural stability (work contracts and benefits), or that is appropriately valued. Green economy interventions often build upon earlier interventions of various kinds, in a “slow burn” of long‐term informality, “stop‐start fashion” or green economy “projectisation”, which mainly operate on three‐ to five‐year timeframes and leave workers in flux as to how any type of livelihood or well‐being can be established (Li 2007). These projects feed off relationships of “coerced collaboration”, where workers have little choice but to take on menial labour tasks in light of the precarious social and economic conditions (Neimark and Wilson 2016). This contributes to the inability of labour to organise—both purposefully structured that way, but also temporally defined through the “slow burn” of informality (Sodikoff 2012). While state and NGO actors may occupy technical roles, the local and often physical labour is contracted out. The state here is then generally absent and has deferred to either civil society and/or “market” actors in the green economy to define and establish labour contracts. Among the categories, several were identified as being more vulnerable/precarious. The geography of precarity in the green economy locates this at the sites of intervention, where voluntary and involuntary action is expected of communities, or labour is deployed away from ongoing livelihood activities to ones that are ultimately uncertain and volatile. Ultimately, precarious labour will contribute to the downfall of the green economy, as REDD+ schemes are already showing, because people have to fall back on the resource use that the schemes are trying to prevent (Milne et al. 2019). While the economic, socio‐ecological and political ramifications have been widely documented, few studies engage with the role of precarious labour which is essential for the standardisation and social abstraction needed for valuation in the green economy (Sodikoff 2007, 2012). More often, critiques of the green economy focus on the notion of participation (Cooke and Kothari 2001; Grove and Pugh 2015; Pasgaard and Nielsen 2016). We suggest that a systematic way is needed to think about labour in the green economy that goes beyond participation.

### What does Resistance Look Like for Precarious Labour in the Green Economy and What are the Implications?

To many workers, the green economy is quite amorphous, consisting of all too familiar discourse of participation and development and a disparate group of NGOs and their donor organisations who manage market‐based interventions. The most recognisable entity, the state, for the most part is either visibly absent or intent on tapping green investments for foreign revenue through overseas development assistance, carbon capture, development, conservation for debt‐relief, farming and fishing. In these contexts, the green economy can become an extension of state power in the face of several decades of deregulation that have given rise to green economy institutions (Corson et al. 2013; Peluso and Lund 2011).

The green economy is in a sense faceless and, much like the precarious labour that underpins it lacks any visible market or state apparatus to resist (Standing 2012). This is why there is sometimes quite subtle resistance (Scott 1985) against the NGOs complicit in the structural precarity of green economy work in the face of “coerced collaboration". The green economy’s precarious labour conditions also tend to thrive in places without accountable institutions and where real political discussion and discourse are absent (Asher and Wainwright 2018; Barca 2019; Mouffe 2016). This has implications particularly today as diverse populist movements are beginning to shape up and organise across the political spectrum from Standing Rock to *gilets jaunes* (Huff 2019). More generally, those politically aligned with labour movements welcome such acts of resistance and dissent, particularly in less observed rural areas and from peasant movements. However, they are also ripe for co‐option, such as in South Africa and India, where farmers' grievances can be picked up by those already in power and used to progress malicious ends and potentially against workers' long‐term interests (Scoones et al. 2018).

Now more than ever we need to recognise labour as central to understanding the uneven development of green economy interventions in resource frontiers. This paper helps do just that, especially for those concerned with geographies of labour and precarity, and other critical scholars seeking to look behind the rhetorical cloak of participation and inclusivity in green economy discourse and policy. We have highlighted that a highly precarious class may be emerging (often from existing power asymmetries) in such interventions. Our hope is for more scholarly attention to understand the precarity and marginalisation potentially created by this green division of labour and the emerging political debates and differing forms of resistance now that seem to be proliferating throughout the global South.

## Endnotes

1 Participatory schemes emerged in the late 1960s and 1970s as a reaction to expert‐led top‐down policies which did not account for local concerns (Chambers 1983).

2 By using the “eco” signifier, we are highlighting work conducted under green economy platforms mainly in the global South, yet as noted above comparisons can be made with precarious workers in service‐based economies globally.

3 These interview data were collected by one co‐author in Palawan, Philippines, May–June 2011, under an ARC Discovery grant.

4 Büscher and Davidov (2013) call this close coupling of green labour and extraction “the ecotourism–extraction nexus”.

5 For exceptions, see Hiraldo Lopez‐Alonso (2017) and Sodikoff (2012).

6
http://www.unwomen.org/en/news/stories/2012/6/fast-forwarding-women-s-leadership-in-the-green-economy (last accessed 15 January 2018).

7
https://www.unenvironment.org/explore-topics/green-economy/about-green-economy (last accessed 8 March 2018).

## References

[anti12593-bib-0001] Adams S and Neumark D (2004) The economic effects of living wage laws: A provisional review. Urban Affairs Review 40(2):210–245

[anti12593-bib-0002] Adams W M (2017) Sleeping with the enemy? Biodiversity conservation, corporations, and the green economy. Journal of Political Ecology 24(1):200–341

[anti12593-bib-0003] Agrawal A and Ribot J C (2000) Analyzing Decentralization: A Framework with South Asian and West African Environmental Cases. Washington, DC: World Resources Institute

[anti12593-bib-0004] Apostolopoulou E and Adams W M (2015) Neoliberal capitalism and conservation in the post‐crisis era: The dialectics of “green” and “un‐green” grabbing in Greece and the UK. Antipode 47(1):15–35

[anti12593-bib-0005] Asher K and Wainwright J (2018) After post‐development: On capitalism, difference, and representation. Antipode 51(1):25–44

[anti12593-bib-0006] Auerbach N and Negi R (2009) Primitive accumulation, capitalism, and development. Human Geography 2(3):100–103

[anti12593-bib-0007] Barca S (2019) The labor(s) of degrowth. Capitalism Nature Socialism 30(2):207–216

[anti12593-bib-0009] Buck D (2009) On primitive accumulation and its shadowy twin, subsumption. Human Geography 2(3):97–99

[anti12593-bib-0012] Büscher B and Davidov V (eds) (2013) The Ecotourism‐Extraction Nexus: Political Economies and Rural Realities of (Un)comfortable Bedfellows. New York: Routledge

[anti12593-bib-0013] Butler J (2006) Precarious Life: The Powers of Mourning and Violence. London: Verso

[anti12593-bib-0014] Carswell G and De Neve G (2013) Labouring for global markets: Conceptualising labour agency in global production networks. Geoforum 44:62–70

[anti12593-bib-0015] Castree N (2007) Labour geography: A work in progress. International Journal of Urban and Regional Research 31(4):853–862

[anti12593-bib-0016] Castree N , Coe N M , Ward K and Samers M (2004) Spaces of Work: Global Capitalism and the Geographies of Labour. London: Sage

[anti12593-bib-0017] Chambers R (1983) Rural Development: Putting the Last First. Harlow: Prentice Hall

[anti12593-bib-0018] Choonara E (2011) Is there a precariat? Socialist Review October http://socialistreview.org.uk/362/there-precariat (last accessed 14 November 2019)

[anti12593-bib-1011] Cleaver F (2012) Development Through Bricolage Rethinking Institutions for Natural Resource Management. NEw York: Routledge

[anti12593-bib-0019] Coe N M (2013) Geographies of production III: Making space for labour. Progress in Human Geography 37(2):271–284

[anti12593-bib-0020] Cooke B and Kothari U (eds) (2001) Participation: The New Tyranny? London: Zed

[anti12593-bib-0021] Conservation International, Province of Palawan, Department of Environment and Natural Resources, Palawan Council for Sustainable Development, and South Palawan Planning Council (2008) “Estimation of the Total Economic Value of the Proposed Mt Mantalingahan Protected Landscape.”

[anti12593-bib-0022] Corson C , MacDonald K I and Neimark B (2013) Grabbing “green”: Markets, environmental governance, and the materialization of natural capital. Human Geography 6(1):1–15

[anti12593-bib-0023] Davidson N (2017) Uneven and combined development: Modernity, modernism, revolution (2)—Causes, consequences, constraints. Revolutionary Socialism in the 21st Century 10 February https://www.rs21.org.uk/2017/02/10/revolutionary-reflections-uneven-and-combined-development-modernity-modernism-revolution-2-causes-consequences-constraints/ (last accessed 15 January 2018)

[anti12593-bib-1002] Doron A and Jeffery R (2018) Waste of a Nation: Garbage and Growth in India. Cambridge, MA: Harvard University Press

[anti12593-bib-1004] Dressler W , Büscher B , Schoon M , Brockington D , Hayes T , Kull C A , McCarthy J and Shrestha K (2010) From hope to crisis and back again? A critical history of the global CBNRM narrative. Environmental Conservation 37(1):5–15

[anti12593-bib-0024] Dressler W H , Smith W and Montefrio M J F (2018) Ungovernable? The vital natures of swidden assemblages in an upland frontier. Journal of Rural Studies 61:343–354

[anti12593-bib-0025] Dressler W H , Wilson D , Clendenning J , Cramb R , Keenan R , Mahanty S , Bruun T , Mertz O and Lasco R D (2017) The impact of swidden decline on livelihoods and ecosystem services in Southeast Asia: A review of the evidence from 1990 to 2015. Ambio 46(3):291–310 27854070 10.1007/s13280-016-0836-z PMC5347523

[anti12593-bib-1005] Elmhirst R (2011) Introducing new feminist political ecologies. Geoforum 42(2):129–132

[anti12593-bib-1010] Elmhirst R (2017) Understories of the political forest: A mobile feminist political ecology? Singapore Journal of Tropical Geography 39(1):41–44

[anti12593-bib-0026] FAO (2014) Asia and the Pacific’s Blue Growth Initiative. Rome: Food and Agriculture Organization of the United Nations

[anti12593-bib-0027] Felli R (2014) On climate rent. Historical Materialism 22(3/4):251–280

[anti12593-bib-0028] Ferguson J and Li T M (2018) “Beyond the ‘Proper Job’: Political‐Economic Analysis after the Century of Labouring Man.” Working Paper 51, Institute for Poverty, Land, and Agrarian Studies (PLAAS)

[anti12593-bib-0029] Fletcher R , Dressler W H , Anderson Z R and Büscher B (2019) Natural capital must be defended: Green growth as neoliberal biopolitics. Journal of Peasant Studies 46(5):1068–1095

[anti12593-bib-1000] Fredericks R (2018) Garbage Citizenship: Vital Infrastructures of Labor in Dakar, Senegal. Durham, NC: Duke University Press

[anti12593-bib-0031] Froese R and Pauly D (2015) “FishBase.” http://www.fishbase.org/search.php (last accessed 15 January 2017)

[anti12593-bib-0032] Gill R and Pratt A (2008) In the social factory? Immaterial labour, precariousness, and cultural work. Theory, Culture, and Society 25(7/8):1–30

[anti12593-bib-0033] Government of Kenya (1994) Kenya Forestry Master Plan. Nairobi: Ministry of Environment and Natural Resources

[anti12593-bib-0034] Grove K and Pugh J (2015) Assemblage thinking and participatory development: Potentiality, ethics, biopolitics. Geography Compass 9(1):1–13

[anti12593-bib-0035] Harvey D (2004) The New Imperialism. Oxford: Oxford University Press

[anti12593-bib-0036] Herod A (1997) From a geography of labor to a labor geography: Labor’s spatial fix and the geography of capitalism. Antipode 29(1):1–31

[anti12593-bib-0037] Hickel J and Kallis G (2019) Is green growth possible? New Political Economy 10.1080/13563467.2019.1598964

[anti12593-bib-0038] Hiraldo Lopez‐Alonso R (2017) Value is labour: Producing environmental rent and commodities for nature tourists in rural Senegal. Human Geography 10(2):54–71

[anti12593-bib-0039] Huff P (2019) Headless populism and the political ecology of alienation. Undisciplined Environments 10 January https://undisciplinedenvironments.org/2019/01/10/the-yellow-vests-a-headless-revolt-against-alienation/ (last accessed 14 November 2019)

[anti12593-bib-0040] Kairu A , Upton C , Huxham M , Kotut K , Mbeche R and Kairo J (2018) From shiny shoes to muddy reality: Understanding how meso‐state actors negotiate the implementation gap in participatory forest management. Society and Natural Resources 31(1):74–88

[anti12593-bib-1008] Kenney‐Lazar M and Kay K (2017) Value in capitalist natures. Capitalism Nature Socialism 28(1):33–38

[anti12593-bib-1003] Knapp F L (2016) The birth of the flexible mine: Changing geographies of mining and the e‐waste commodity frontier. Environment and Planning A: Economy and Space 48(10):1889–1909

[anti12593-bib-0041] Lambert R and Herod A (eds) (2016) Neoliberal Capitalism and Precarious Work: Ethnographies of Accommodation and Resistance. Cheltenham: Edward Elgar

[anti12593-bib-0042] Lansing D M (2012) Performing carbon’s materiality: The production of carbon offsets and the framing of exchange. Environment and Planning A 44(1):204–220

[anti12593-bib-1001] Lawhon M , Millington N and Stokes K (2018) A labour question for the 21st century: Perpetuating the work ethic in the absence of jobs in South Africa's waste sector. Journal of Southern African Studies 44(6):1115–1131

[anti12593-bib-0043] Lewis H , Dwyer P , Hodkinson S and Waite L (2015) Hyper‐precarious lives: Migrants, work, and forced labour in the global North. Progress in Human Geography 39(5):580–600

[anti12593-bib-0044] Li T M (2007) The Will to Improve: Governmentality, Development, and the Practice of Politics. Durham: Duke University Press

[anti12593-bib-0046] Li T M (2016) Governing rural Indonesia: Convergence on the project system. Critical Policy Studies 10(1):79–94

[anti12593-bib-0047] Li T M (2017) After development: Surplus population and the politics of entitlement. Development and Change 48(6):1247–1261

[anti12593-bib-1012] Lovell H and Ghaleigh N S (2013) Climate change and the professions: The unexpected places and spaces of carbon markets. Transactions of the Institute of British Geographers 38(3):512–516

[anti12593-bib-0048] Lovell H and MacKenzie D (2011) Accounting for carbon: The role of accounting professional organisations in governing climate change. Antipode 43(3):704–730

[anti12593-bib-0049] MacDonald K I (2010) The devil is in the (bio) diversity: Private sector “engagement” and the restructuring of biodiversity conservation. Antipode 42(3):513–550

[anti12593-bib-0050] Mahanty S , Bradley A and Milne S (2015) The forest carbon commodity chain in Cambodia’s voluntary carbon market In Milne S and Mahanty S (eds) Conservation and Development in Cambodia: Exploring Frontiers of Change in Nature, State, and Society (pp 177–200). Abingdon: Routledge

[anti12593-bib-0051] Marx K (2011 [1867]) Capital, Volume One: A Critique of Political Economy (trans S Moore and E Aveling; ed Engels F). Mineola: Dover

[anti12593-bib-0052] Meehan K and Stauss K (eds) (2015) Precarious Worlds: Contested Geographies of Social Reproduction. Athens: University of Georgia Press

[anti12593-bib-0053] Mertz O , Leisz S J , Heinimann A , Rerkasem K , Dressler W , Pham V , Vu K , Schmidt‐Vogt D , Colfer C , Epprecht M , Potter L and Padoch C (2009) Who counts? Demography of swidden cultivators in Southeast Asia. Human Ecology 37(3):281–289

[anti12593-bib-0054] Mills D J , Westlund L , de Graaf G , Kura Y , Willman R and Kelleher K (2011) Under‐reported and undervalued: Small‐scale fisheries in the developing world In Pomeroy R S and Andrews N (eds) Small‐Scale Fisheries Management: Frameworks and Approaches for the Developing World (pp 1–15). Wallingford: CABI

[anti12593-bib-0056] Milne S and Mahanty S (2019) Value and bureaucratic violence in the green economy. Geoforum 98:133–143

[anti12593-bib-0057] Milne S , Mahanty S , To P , Dressler W , Kanowski P and Thavat M (2019) Learning from “actually existing” REDD+: A synthesis of ethnographic findings. Conservation and Society 17(1):84–95

[anti12593-bib-0058] Moore J W (2015) Capitalism in the Web of Life: Ecology and the Accumulation of Capital. London: Verso

[anti12593-bib-0059] Mouffe C (2016) The populist movement. OpenDemocracy 21 November https://www.opendemocracy.net/democraciaabierta/chantal-mouffe/populist-moment (last accessed 17 March 2018)

[anti12593-bib-0060] Munck R (2013) The precariat: A view from the South. Third World Quarterly 34(5):747–762

[anti12593-bib-0061] Neimark B , Childs J , Nightingale A J , Cavanagh C J , Sullivan S , Benjaminsen T A , Batterbury S , Koot S and Harcourt W (2019) Speaking power to “post‐truth”: Critical political ecology and the new authoritarianism. Annals of the Association of American Geographers 109(2):613–623

[anti12593-bib-0062] Neimark B , Mahanty S and Dressler W (2016) Mapping value in a “green” commodity frontier: Revisiting commodity chain analysis. Development and Change 47(2):240–265

[anti12593-bib-0063] Neimark B D and Vermeylen S (2017) A human right to science? Precarious labor and basic rights in science and bioprospecting. Annals of the American Association of Geographers 107(1):167–182

[anti12593-bib-0064] Neimark B D and Wilson B (2015) Re‐mining the collections: From bioprospecting to biodiversity offsetting in Madagascar. Geoforum 66:1–10

[anti12593-bib-0065] Neumann R P (1998) Imposing Wilderness: Struggles over Livelihood and Nature Preservation in Africa. Berkeley: University of California Press

[anti12593-bib-0067] Pasgaard M and Nielsen T F (2016) A story of “communities”: Boundaries, geographical composition, and social coherence in a forest conservation project, Northern Cambodia. Geografisk Tidsskrift—Danish Journal of Geography 116(2):134–146

[anti12593-bib-0068] Peluso N L and Lund C (2011) New frontiers of land control. Journal of Peasant Studies 38(4):667–681

[anti12593-bib-0069] Protected Area Management Board (2010) “Mt Mantalingahan Protected Landscape Management Plan.”

[anti12593-bib-0070] Räthzel N and Uzzell D (eds) (2012) Trade Unions in the Green Economy: Working for the Environment. Abingdon: Routledge

[anti12593-bib-0071] Robertson M (2012) Measurement and alienation: Making a world of ecosystem services. Transactions of the Institute of British Geographers 37(3):386–401

[anti12593-bib-0072] Rönnbäck P , Crona B and Ingwall L (2007) The return of ecosystem goods and services in replanted mangrove forests: Perspectives from local communities in Kenya. Environmental Conservation 34(4):313–324

[anti12593-bib-0073] Ross A (2008) The new geography of work: Power to the precarious? Theory, Culture, and Society 25(7/8):31–49

[anti12593-bib-0074] Saatchi S S , Harris N L , Brown S , Lefsky M , Mitchard E T , Salas W , Zutta B R , Buerman W , Lewis S L , Hagen S , Petrova S , White L , Silman M and Morel A (2011) Benchmark map of forest carbon stocks in tropical regions across three continents. Proceedings of the National Academy of Sciences 108(24):9899–9904 10.1073/pnas.1019576108 PMC3116381 21628575

[anti12593-bib-0075] Sandbrook C , Luque‐Lora R and Adams W M (2018) Human bycatch: Conservation surveillance and the social implications of camera traps. Conservation and Society 16(4):493–504

[anti12593-bib-0076] Scoones I , Edelman M , Borras Jr S M , Hall R , Wolford W and White B (2018) Emancipatory rural politics: Confronting authoritarian populism. Journal of Peasant Studies 45(1):1–20

[anti12593-bib-0077] Scott J C (1985) Weapons of the Weak: Everyday Forms of Peasant Resistance. New Haven: Yale University Press

[anti12593-bib-0078] Siegmann K A and Schiphorst F (2016) Understanding the globalizing precariat: From informal sector to precarious work. Progress in Development Studies 16(2):111–123

[anti12593-bib-0079] Sklair L (2001) The Transnational Capitalist Class. Oxford: Blackwell

[anti12593-bib-0080] Smith N (2007) Nature as accumulation strategy In Panitch L and Leys C (eds) Socialist Register 2007: Coming to Terms with Nature (pp 16–36). London: Merlin

[anti12593-bib-0081] Sodikoff G (2007) An exceptional strike: A micro‐history of “people versus park” in Madagascar. Journal of Political Ecology 14(1):10–33

[anti12593-bib-0082] Sodikoff G (2012) Forest and Labor in Madagascar: From Colonial Concession to Global Biosphere. Bloomington: Indiana University Press

[anti12593-bib-0083] Standing G (2009) Work after Globalisation: Building Occupational Citizenship. Cheltenham: Edward Elger

[anti12593-bib-1006] Standing G (2011) The Precariat: The New Dangerous Class. London and New York: Bloomsbury Academic

[anti12593-bib-0084] Standing G (2012) The precariat: From denizens to citizens? Polity 44(4):588–608

[anti12593-bib-0085] Standing G (2014) The precariat: The new dangerous class. Amalgam 6(6/7):115–119

[anti12593-bib-0086] Strauss K (2013) Unfree again: Social reproduction, flexible labour markets, and the resurgence of gang labour in the UK. Antipode 45(1):180–197

[anti12593-bib-0087] Strauss K (2018) Labour geography II: Being, knowledge, and agency. Progress in Human Geography 10.1177/0309132518803420

[anti12593-bib-0088] Thompson P (1989) The Nature of Work: An Introduction to Debates on the Labour Process. London: Macmillan

[anti12593-bib-0089] Tollefson J (2016) Paris climate deal hinges on better carbon accountancy. Nature News 529(7587):450–451 10.1038/529450a 26819026

[anti12593-bib-0090] Tsing A L (2000) Inside the economy of appearances. Public Culture 12(1):115–144

[anti12593-bib-0091] Urry J (2014) Offshoring. Hoboken: John Wiley and Sons

[anti12593-bib-1007] Waite L , Dwyer P , Hodkinson S and Lewis H (2013) Precarious Lives: Forced Labour, Exploitation and Asylum. Bristol: Policy Press

[anti12593-bib-0092] Werner M (2015) Global Displacements: The Making of Uneven Development in the Caribbean. Hoboken: John Wiley and Sons

[anti12593-bib-0093] West P (2006) Conservation is Our Government Now: The Politics of Ecology in Papua New Guinea. Durham: Duke University Press

[anti12593-bib-0094] Wolf E R (2010) Europe and the People Without History. Berkeley: University of California Press

[anti12593-bib-0095] World Bank (2019) World Development Report 2019: The Changing Nature of Work. Washington, DC: World Bank

[anti12593-bib-0096] Yeoh B S and Huang S (1998) Negotiating public space: Strategies and styles of migrant female domestic workers in Singapore. Urban Studies 35(3):583–602

